# Estimation of Genetic Parameters for Body Weight and Its Stability in Huaxi Cows from Xinjiang Region

**DOI:** 10.3390/ani15152248

**Published:** 2025-07-31

**Authors:** Ye Feng, Wenjuan Zhao, Xubin Lu, Xue Gao, Qian Zhang, Bin Zhang, Bao Wang, Fagang Zhong, Mengli Han, Zhi Chen

**Affiliations:** 1 College of Animal Science and Technology, Yangzhou University, Yangzhou 225009, China; mz120241624@stu.yzu.edu.cn (Y.F.); 008212@yzu.edu.cn (X.L.); 2 State Key Laboratory for Sheep Genetic Improvement and Healthy Production, Xinjiang Academy of Agricultural and Reclamation Science, Shihezi 832000, China; zwj-130@163.com (W.Z.); 15299909121@163.com (Q.Z.); binzhangth@163.com (B.Z.); 13579707588@163.com (B.W.); zfg125@sohu.com (F.Z.); 3 Cattle Genetics and Breeding Group, Institute of Animal Science (IAS), Chinese Academy of Agricultural Sciences (CAAS), Beijing 100193, China; gaoxue76@126.com

**Keywords:** adult weight, birth weight, heritability, genetic analysis, West China cattle

## Abstract

In this study, we analyzed data from 2992 cows to comprehensively evaluate the adult weight (WEI), a key growth and body-size indicator, in West China cattle, aiming to estimate the related phenotypic and genetic parameters. The analysis focused on four weight traits while considering non-genetic factors such as parity, season, year, and birth weight. Data were processed and corrected using a MIXED procedure and a multi-trait animal model. Results showed that these non-genetic factors significantly affected the weight traits (*p* < 0.05), which had high heritability (0.25–0.39) (*p* < 0.01). WEI is crucial for improving the genetic traits of cattle in western China and provides innovative approaches for optimizing herd management, enhancing the efficiency of genetic selection, and boosting beef cattle productivity.

## Simple Summary

This study focuses on evaluating the phenotypic and genetic traits of adult weight in 2992 Huaxi dairy cows, proposing a weight-evaluation metric named WEI. Calculated from birth, 6-month, 12-month, and adult weights, WEI covers the entire growth cycle. The MIXED procedure assessed the impacts of non-genetic factors such as parity, season, birth year, and birth weight on adult weight, and they were found to be significantly correlated. With a value of 0.25–0.39, adult weight shows high heritability. WEI is of significant importance to beef cattle breeding in western China, offering new perspectives for herd management and genetic selection and contributing to an enhancement in beef production efficiency.

## 1. Introduction

Huaxi cattle, a specialized meat breed independently developed in China in 1978, originated from crossbreeding beef-type Simmental, Mongolian, Sanhe, and Charolais cattle [1]. As a result of 43 years of crossbreeding and selection, this breed now exhibits a large body size and fast growth rate. Mature bulls weigh about 900 kg, and cows weigh about 550 kg [2]. The average daily weight gain for fattening cattle aged 12–18 months is approximately 1.36 kg, and they also have high slaughter and net meat rates [3]. Widely adaptable, the Huaxi cattle thrive in both stall-feeding in farming areas and grazing plus supplementary feeding in pastoral regions [4]. Huaxi cattle breeding has been promoted in multiple provinces, including Inner Mongolia, Jilin, and Henan. By 2024, national core breeding groups had reached a scale of 23,400, with the number of bull stations rising from 3 to 25, and the number of provinces promoting the breed increasing from 6 to 12. Over 64% of the national core and breeding groups are located in key breeding bases such as Xilin Gol League [5].

In beef cattle breeding, meat quality is a key economic trait and is highly weighted in breeding goals globally [6]. Over the past century, selection for meat yield has undergone significant genetic improvement. National beef cattle associations routinely collect weight data for genetic evaluation of adult body weight. These data-driven measurements support the selection of the trait, and intensive genetic selection has generated substantial genetic gains in the field [7].

The widespread use of electronic weighing and automated weight-monitoring systems has made it possible to collect large amounts of precise longitudinal weight data, transforming the phenotyping of beef cattle growth traits. New indices such as the weight-fluctuation resilience based on continuous monthly weight records are now emerging as frontiers in beef cattle breeding research [8]. Adult body weight in beef cattle refers to the weight when they reach physiological maturity and stop growing. This vital growth-performance indicator reflects cattle body size and growth potential, playing a crucial role in evaluating growth and body size within beef cattle husbandry [9]. The weight-gain rate, calculated from monthly weight records as the average daily weight gain, is a key indicator of growth potential. It is used not only for optimizing feeding management but also for analyzing disease-occurrence risks [10]. Compared to single-time-point weight measurements, the weight-gain rate provides a more comprehensive and accurate assessment of growth performance. This not only offers valuable decision-making support for breeders and feeders but, more importantly, helps them pinpoint the factors behind weight fluctuations. Moreover, the stability of the weight-gain rate indicates cattle’s adaptability to environmental changes [11]. By analyzing its standard deviation and range, weight stability can be assessed at different growth stages, presenting new ideas for breeding and likely becoming a key consideration in future breeding research. In genetic research, environmental effects are also an indispensable component, influencing phenotypic traits as well as genetic parameters [12,13]. In past research, genetic parameters for adult body weight in beef cattle have been widely studied, with heritability reported to range from 0.1 to 0.4. However, phenotypic and genetic research on adult body weight in Huaxi cows is limited. In this study, we conducted phenotyping and genetic analysis using records from a large dairy farm to estimate phenotypic and genetic parameters, simultaneously considering the impact of environmental effects on body weight. Our findings not only address a research gap but also offer theoretical and practical guidance for cattle management and the genetic selection of Huaxi cows’ production performance, advancing the field of breeding research.

## 2. Materials and Methods

### 2.1. Raw Data

In this study, we collected detailed weight records of 2992 Huaxi cows from a large-scale cattle farm in Xinjiang, China, spanning from January 2020 to July 2024. On this farm, standardized breeding practices are implemented, with free access to clean water and well-formulated TMR for different growth stages. TMR (Total Mixed Ration) is a nutritionally balanced feed formulated through precise ingredient proportioning and standardized processing. Its mixing protocol strictly adheres to animal nutritional requirement models, ensuring every ration meets the nutritional needs of specific production phases. The TMR formulation and nutritional parameters for Huaxi cows at different growth stages are as follows (on a dry matter basis): For the calf stage, the formulation consists of corn (40–45%), corn silage (25–30%), soybean meal (15–18%), and mineral premix (2%). The nutritional indicators include 11.8–12.2 MJ/kg of metabolizable energy, 18.0–20.5% of crude protein, 28–32% of neutral detergent fiber, 0.70–0.85% of calcium, 0.40–0.48% of phosphorus, and 8000–10,000 IU/kg of vitamin A. For the juvenile stage, the formulation comprises corn (50–55%), corn silage (30–35%), rapeseed meal (12–15%), dicalcium phosphate (1.5%), and salt (0.5%). The nutritional indicators are 12.2–12.8 MJ/kg of metabolizable energy, 16.0–17.8% of crude protein, 20–24% of acid detergent fiber, 0.80–0.95% of calcium, and 0.45–0.52% of phosphorus. For the adult lactating stage, the formulation includes corn (45–50%), whole plant silage (40–45%), soybean meal (10–12%), corn gluten meal (5–8%), calcium carbonate (1.2%), and bentonite (1.0%). The nutritional indicators are 12.5–13.2 MJ/kg of metabolizable energy, 17.5–19.2% of crude protein, ≥60% of rumen degradable protein, 0.90–1.15% of calcium, 0.48–0.58% of phosphorus, and 0.28–0.32% of magnesium. For the dry cow stage, the formulation is composed of corn (35–40%), oat hay (20–25%), corn silage (25–30%), cottonseed meal (8–10%), and premix (2.5%). The nutritional indicators are 10.2–10.8 MJ/kg of metabolizable energy, 12.5–14.0% of crude protein, 0.50–0.62% of calcium, and 0.30–0.38% of phosphorus. In the adjusted plan for high-yielding cows, the concentrate proportion is increased by 15–20%, 30% of cottonseed meal is replaced by soybean meal, and 3–5% rumen-protected fat is added. The nutritional indicators are as follows: ≥13.0 MJ/kg of metabolizable energy, ≥18.5% of crude protein, 5.5–6.5% of fat content, and 1.05–1.25% of calcium. These tailored TMR formulations enhance production performance and health. The original dataset contains 11,972 weight-related data points, recording individual and phenotypic information such as birth season, parity, withers height, body weight, chest girth, body length, height at the cross, cannon bone girth, and abdominal girth. In this study, Xinjiang Agricultural University collaborated with the locally based Huaxi Cattle Farm in Xinjiang to collect and provide raw pedigree data. Researchers traced the pedigree of each Huaxi cow with phenotypic records, achieving an average tracing depth of five generations to ensure the completeness and accuracy of the pedigree information. The pedigree database for genetic analysis comprised 296 bulls and 3595 cows, totaling 3891 Huaxi cattle. This large-scale and detailed pedigree database offers a robust foundation for subsequent genetic evaluation and analysis, significantly enhancing the accuracy and credibility of the research findings.

### 2.2. Data Quality Control and Trait Definition

To ensure data reliability, we systematically integrated raw data from Xinjiang Agricultural University and partner dairy farms, verifying comprehensive coverage of core fields—birth season, year, parity, weight, chest girth, and body length—with unified formats. During data cleaning, a tiered missing-value strategy was implemented: records missing key identifiers (e.g., ear tags) were deleted immediately; entries lacking core analytical variables (parity, birth year) were removed when reliable inference proved impossible; entire records with >30% missing core fields were discarded; and numerical variables (weight/chest girth/body length) with <5% missing rates were imputed using breed-specific means (e.g., replacing missing Huaxi cattle weight with the breed average). Duplicate records were resolved by retaining the latest or most complete entries based on ear tag identifiers, while numerical outliers underwent dual-channel detection via boxplots and Z-scores (preliminary threshold |Z| > 3), followed by manual verification against preset biologically plausible ranges (e.g., physiological weight intervals). Erroneous, uncorrectable entries were deleted, whereas biologically valid extremes were preserved with annotations. Data were validated through three-pronged verification: examining biological logic (e.g., weight–age progression), ensuring cross-record consistency for individual cows (e.g., birth date–parity alignment), and confirming unit standardization. Subsequent processing featured Z-score standardization for continuous variables and one-hot/label encoding for categorical variables, with quality assessed through statistical metrics (mean/SD), correlation analyses, and scatter/boxplot visualizations. A final quality control report consolidated findings to support genetic analysis and modeling, with Table 1 detailing record counts at each filtration stage (e.g., missing, duplicates, biologically implausible values).

In this study, the heritability coefficient of adult body weight was calculated based on birth, 6-month, 12-month, and adult weights, reflecting their impact on adult weight. Given the 6-month weighing interval, heritability was calculated separately for birth weight (WEI-0), 6-month weight (WEI-6), and 12-month weight (WEI-12), as well as for each weighing interval. The effects of parity, birth weight, birth season, and year on weight gain over 6-month intervals were analyzed. Abbreviations and definitions of all analyzed traits are presented in Table 2.

### 2.3. Statistical Analysis

In this study, the blupADC package in Rstudio (version 4.4; 2024) was used to assess the impact of non-genetic factors (parity, measurement season, birth weight, and birth year) on monthly and adult weights. Individual random effects were included in the multi-trait animal model, and multiplicity tests were used for multiple comparisons across levels of each non-genetic factor. Parities were grouped into 5 levels—1, 2, 3, 4, and ≥5—with the last level covering parities 5 to 11. Seasons were categorized into spring (March–May), summer (June–August), autumn (September–November), and winter (December–February) based on Xinjiang’s climate. In constructing the multiple regression model for adult body weight prediction, we systematically evaluated multicollinearity among the predictors (WEI-0, WEI-6, WEI-12, chest circumference, body length). The results demonstrate the following: WEI-0: VIF = 1.08; WEI-6: VIF = 3.42; WEI-12: VIF = 4.15; chest circumference: VIF = 2.67; body length: VIF = 2.13. All VIF values were <5, indicating no substantial multicollinearity requiring correction in our model.

The DMU automatic analysis model (version 2021.8.24) estimates variance and covariance components for monthly and adult weights, considering complex factors such as maternal effects, permanent environmental effects, and random regression. Multi-trait animal models accurately estimate heritability and repeatability for each trait. A linear mixed-effects model combining fixed and random effects analyzes the impact of multiple factors on weight. This approach suits complex biological data with genetic and environmental components. These models also enable estimation of genetic correlations among birth weight, monthly weights, and adult weight. The model for monthly and adult weights is as follows:

(1) $$W_{ijklmn\;=\;}{YEAR}_{i}+{SEASON}_{j}+{PARITY}_{k}+{BIRTH}_{l}+{BW}_{m}+a_{ijklmn}+{pe}_{ijklmn}+e_{ijklmn}$$

Here, *W* is the phenotypic vector for birth weight (WEI-0), weight at 6 months of age (WEI-6), weight at 12 months of age (WEI-12), and adult weight (WEI). ${YEAR}_{i}$ is the vector of fixed effects for the birth year (i = 1, 2,…, 4, representing 2020, 2021, 2022, etc., respectively); ${SEASON}_{j}$ is the vector of fixed effects for the testing season (j = 1, 2,…, 4, representing spring, summer, autumn, and winter, respectively); and ${PARITY}_{k}$ is the vector of fixed effects for parity (*k* = 1, 2,…, 5, representing parity 1, parity 2, parity 3, parity 4, and parity 5 and above, respectively). When measuring the monthly-age weight of each individual, the parity remains consistent. ${BIRTH}_{l}$ is the vector of fixed effects for birth weight, and ${BW}_{m}$ is the regression term for birth weight. In model construction, we introduced birth weight both as a categorical variable “*BIRTH*” and a continuous variable “*BW_m_*” to comprehensively capture its complex effects on subsequent traits. Categorizing birth weight into low, medium, and high classes as the categorical variable “*BIRTH*” allows for the precise reflection of its non-linear effects on growth, development, and health status across different ranges. For instance, individuals with low and high birth weights may exhibit significantly different biological characteristics, differences which are difficult to fully reveal using linear regression alone. Concurrently, incorporating birth weight as the continuous variable “*BW_m_*” enables the precise quantification of its linear effect on subsequent traits, clarifying the specific effect of a one-unit increase in birth weight. Although “*BIRTH*” is grouped based on “*BW_m_*”, resulting in some correlation between them, this correlation does not constitute complete linear dependence. Through correlation analysis and Variance Inflation Factor (VIF) testing, we confirmed that the correlation between the two is within acceptable limits and that the model does not suffer from severe multicollinearity issues. Furthermore, compared to simplified models containing only a single variable, the current model incorporating both “*BIRTH*” and “*BW_m_*” demonstrates superior explanatory and predictive power, further validating the rationale and scientific rigor of our model. $a_{ijklmn}$ is the random additive genetic effect, with a∼N(0, ${A\sigma }_{a}^{2}$); ${pe}_{ijklmn}$ is the random permanent environmental effect, with pe∼N(0, ${I\sigma }_{pe}^{2}$); $e_{ijklmn}$ is the random residual effect, with e∼N(0, ${I\sigma }_{pe}^{2}$). Here, A is the additive genetic relationship matrix constructed from the pedigree, $\sigma_{a}^{2}$ is the additive genetic variance, *I* is the identity matrix, $\sigma_{pe}^{2}$ is the permanent environmental variance, and $\sigma_{a}^{2}$ is the residual variance.

Univariate analysis is conducted to estimate heritability ($h^{2}$) and repeatability (t). Heritability ($h^{2}$) is calculated as the ratio of additive genetic variance ($\sigma_{a}^{2}$) to total variance, with the formula $h^{2}$ = $\sigma_{a}^{2}$/($\sigma_{a}^{2}$ + $\sigma_{pe}^{2}$ + $\sigma_{e}^{2}$) (Model 2). Repeatability (t) is determined as the ratio of the sum of genetic variance and permanent environmental variance to total variance, with the formula t = ($\sigma_{a}^{2}$ + $\sigma_{pe}^{2}$)/($\sigma_{a}^{2}$ + $\sigma_{pe}^{2}$ + $\sigma_{e}^{2}$) (Model 3). Here, $\sigma_{a}^{2}$ represents additive genetic variance, $\sigma_{pe}^{2}$ represents permanent environmental variance, and $\sigma_{e}^{2}$ is the residual variance for the corresponding trait.

Heritability refers to the ratio of genetic variance to total variance for a specific trait, indicating the extent to which the trait is influenced by genetic factors. Narrow-sense heritability (*h*^2^) considers only the additive genetic variance (i.e., variance contributed by the additive effects of genes), with its calculation formula as follows:

$$h^{2}=\frac{V_{A}}{V_{P}}$$

Here, $V_{A}$ denotes the variance arising from additive genetic effects, while $V_{P}$ represents the total phenotypic variance that integrates both genetic and environmental factors. This parameter quantifies the proportion of phenotypic variation attributable to additive genetic effects. In genetic breeding research, narrow-sense heritability directly elucidates the contribution mechanism of additive gene effects to phenotypic differentiation and further provides the theoretical basis for breeding value prediction and selection response evaluation.

## 3. Results

### 3.1. Descriptive Statistics

Table 3 presents the descriptive statistics of the traits used for the genetic parameter analysis of Huaxi cattle in this study. The birth weight of Huaxi cattle ranged from 23.80 to 63.87 kg, with a mean of 44.60 kg and a standard deviation (SD) of 6.12 kg. The body weight at 6 months ranged from 140.32 to 360.11 kg, averaging 226.56 kg (SD 27.12 kg). The body weight at 12 months ranged from 213.31 to 483.3 kg, averaging 357.15 kg (SD 28.10 kg). Finally, the adult body weight ranged from 421.32 to 788.10 kg, with a mean of 575 kg (SD 36.11 kg). Adult chest girth ranged from 156 to 250 cm, averaging 202 cm (SD 10.00 cm). Adult body length was between 125 and 296 cm, with a mean of 176 cm (SD 18.00 cm). Adult withers’ height varied from 128 to 240 cm, averaging 148 cm (SD 6.10 cm). Adult cannon circumference ranged from 17 to 24 cm, averaging 21 cm (SD 9.11 cm). Adult abdominal girth was from 155 to 260 cm, averaging 237 cm (SD 1.15 cm).

**Figure 1:**
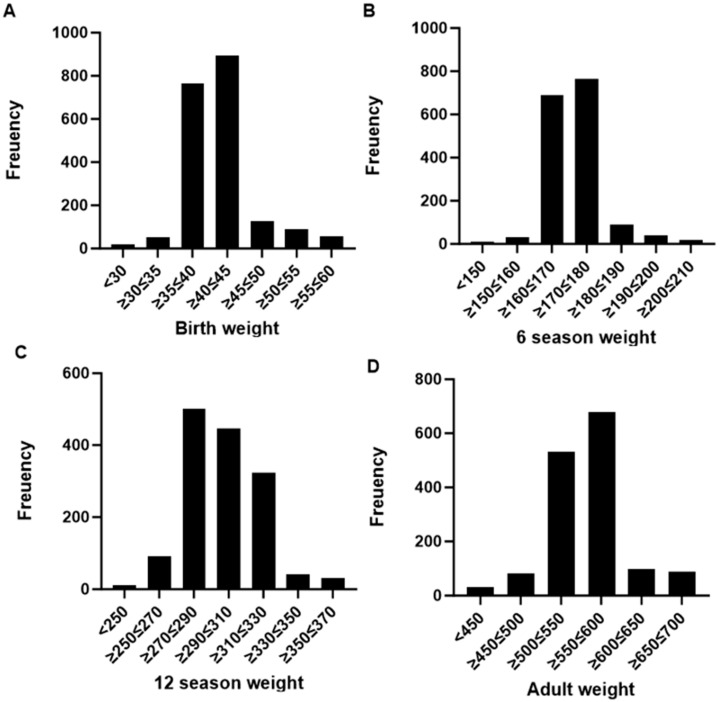
Weight distributions at birth (**A**), 6 months (**B**), 12 months (**C**), and adult (**D**) age. Birth weight: the birth weight of Huaxi cows; 6-month weight: the weight of Huaxi cows at 6 months of age; 12-month weight: the weight of Huaxi cows at 12 months of age; adult weight: the adult weight of Huaxi cattle.

Figure 1 shows the distribution of adult weight and weights at birth, 6 months, and 12 months. The birth weight is mainly concentrated between 35 and 45 kg (SD 6.12 kg), the weight at 6 months is between 160 and 200 kg (SD 27.12 kg), the weight at 12 months is mostly between 260 and 320 kg (SD 28.10 kg), and the adult weight ranges from 500 to 600 kg (SD 36.11 kg).

### 3.2. Impacts of Non-Genetic Effects

This study delved into the key factors affecting the weight development in Huaxi cows and found that parity, measurement season, and birth weight significantly impact the weight at different ages, as well as the adult weight. Statistical analysis shows that for every 1-kg increase in birth weight, the weaning weight can increase by about 2.3 kg and the adult weight can increase by about 3.7 kg. This early growth advantage mainly stems from the inherent advantages of high-birth-weight calves regarding skeletal development and muscle fiber numbers, enabling them to exhibit higher feed conversion rates and growth efficiency in subsequent growth stages.

Table 4 shows the least-squares mean estimates of multiple comparisons for different levels. Specifically, calf birth weight increased with parity, peaked at a certain parity, and then declined. Among seasons, calves born in spring had the highest weight, while those born in summer had the lowest. Moreover, birth weight had a significant impact on age-specific weight. Calves born to cattle in their third to fifth parity were likely to have higher body weights.

### 3.3. Genetic Parameters

The heritability of weight in Huaxi cows is relatively high, with values ranging from 0.25 to 0.40. Table 5 presents the variance components and heritability estimates for age-specific body weight traits in Huaxi cattle. The heritability of 12-month-old body weight is relatively high (h^2^ = 0.399), whereas that of 6-month-old body weight is comparatively low, approximately 0.19. There is a hereditary connection between the monthly weight and adult weight of Huaxi cows, influenced by the continuity of growth and genetic factors. Their growth follows an “S”-shaped curve, with rapid weight gain before 12 months, slowing down afterward.

### 3.4. Correlation Between Measurements and Weight, and Mature Weight Prediction

A comprehensive correlation analysis of Huaxi cattle body weight with multiple measurement indicators revealed significant positive correlations between body weight and morphological traits such as heart girth, body length, and withers height. At birth, the correlation coefficient between body weight and heart girth was as high as 0.83, and with body length, it was 0.79. This indicates that these morphological indicators can effectively reflect the growth status of calves in the early stages and serve as important references for assessing their early growth and development. As the cattle aged, the correlation strengthened. By 6 months of age, the correlation coefficient between weight and heart girth further increased to 0.87, while with body length, it became 0.81. By 12 months of age, the correlations reached higher values, again highlighting that, with increasing age, these morphological indicators become increasingly predictive of body weight and can more precisely depict the growth trajectory of the cattle.

When Huaxi cattle reached adulthood, the correlation coefficients between body weight and heart girth, body length, and withers height were as high as 0.91, 0.89, and 0.83, respectively. These results underscore the significant role and necessity of morphological measurements in assessing adult body weight. Using birth weight, 6-month weight, 12-month weight, and the corresponding heart girth and body length measurements as independent variables, and adult body weight as the dependent variable, a highly accurate prediction model was developed through multiple linear regression analysis. The model revealed that, among all variables, 12-month weight was the most significant contributor to predicting mature weight, with a regression coefficient of 0.67, explaining over 40% of the variation. The regression coefficients for birth weight and 6-month weight were 0.23 and 0.31, respectively, while those for heart girth and body length ranged between 0.15 and 0.25. This prediction model holds substantial reference value for practical production management. On one hand, the model can be used in breeding program formulation to identify individuals with high potential for mature weight, enabling genetic improvement and providing a scientific basis for optimizing the Huaxi cattle breed. On the other hand, feeding strategies can be planned in advance based on the model’s predictions. This allows for rational adjustments to feed supply and stocking density, optimizing resource allocation, and consequently improving overall production efficiency. This approach contributes to advancing the scientific and precise development of the Huaxi cattle breeding industry.

Genetic correlation coefficients (ranging from −1 to +1) measure the genetic associations between body weights at different ages and between body weight at specific ages and adult body weight in Huaxi cows. Table 6 shows an extremely high genetic correlation (0.99) between body weight at 6 months and body weight at 12 months, while the genetic correlation between body weight at 12 months and adult body weight is significantly lower (approximately 0.089). These data are highly valuable for understanding the genetic patterns of body weight traits in Huaxi cows and can effectively inform genetic evaluation and breeding decisions.

**Figure 2:**
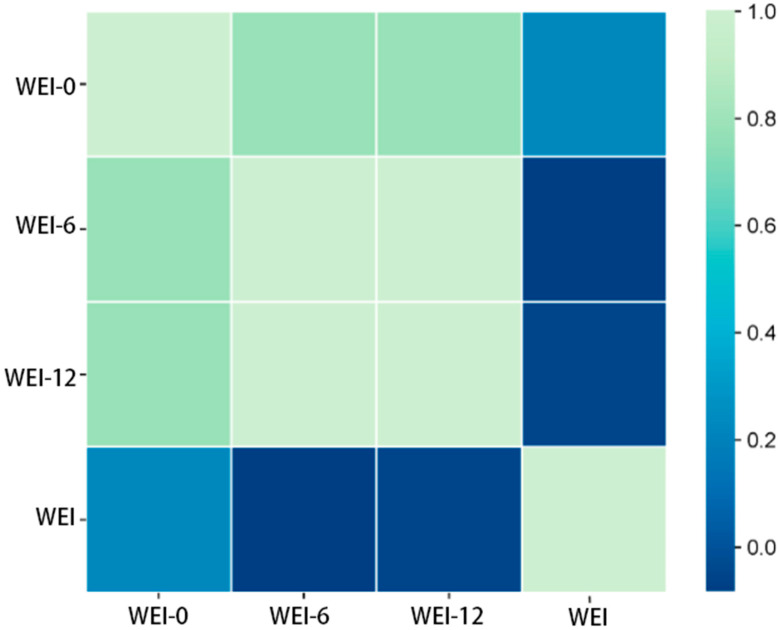
Genetic correlations between weights at different ages and between age-specific weights and adult weight. WEI, adult weight; WEI-0, birth weight; WEI-6, weight at 6 months of age; WEI-12, weight at 12 months of age.

Figure 2 illustrates the distribution of genetic correlations between weights at different ages and between age-specific weights and adult weight. It shows that genetic correlation is strongest between 6- and 12-month weights and weakest between 12-month weight and adult weight.

## 4. Discussion

Genetic covariance reveals the degree of covariation among quantitative traits caused by genetic factors, particularly additive genetic effects, and is crucial for understanding genetic associations between traits and gene pleiotropy and for guiding breeding practices [14,15]. Its estimation requires utilizing phenotypic data from related individuals and employing statistical methods (such as variance and correlation analyses) to distinguish between genetic contributions and environmental influences [16]. This study focused on the genetic characteristics of body weight traits in Huaxi cattle, estimating genetic parameters for weights at different developmental stages (birth weight, WEI-0; 6-month weight, WEI-6; 12-month weight, WEI-12) and mature weight (WEI) [17]. The results revealed high heritability values for these traits (ranging from 0.25 to 0.39), along with significant genetic correlations within the normal range (−1 to +1) both between weights at different stages and between each stage-specific weight and mature weight [18]. This indicates that individuals exhibiting superior early growth performance generally maintain a larger body size in adulthood, reflecting a growth pattern predominantly governed by genetic mechanisms: gene combinations and their variants influence weight-gain efficiency through stage-specific regulation, underpinned by a shared genetic regulatory network across multiple stages [19]. Based on genome-wide association studies (GWASs), we identified stage-specifically expressed genes regulating body weight (e.g., genes primarily governing early development, such as at 6 months, or regulating later growth stages, such as from 12 months to maturity) [20]. This research not only systematically elucidates the genetic architecture of body weight traits in Huaxi cattle (encompassing heritability, genetic correlations, and key genes) but also provides a solid theoretical foundation for establishing a marker-assisted precision breeding system, directly contributing to the enhancement of production performance and economic returns [21,22]. However, it should be noted that the sample size of 2992 cattle presents statistical constraints, which pose limitations in the precision of genetic parameter estimates (such as potential overestimation of heritability). Given genetic and environmental differences, these results might not fully apply to all Huaxi cows in China or other regions [23]. Future research with larger, more diverse samples is needed to enhance the representativeness of genetic parameter estimates and broaden their applicability [24]. Genetic parameters serve as the cornerstone for deciphering the genetic architecture of target traits and optimizing breeding programs. Among these, the heritability coefficient characterizes the proportion of additive genetic variance to phenotypic variance, quantifying the extent to which genetic factors govern the expression of specific traits [25]. Precision breeding based on multi-trait genetic evaluation models (such as BLUP), through breeding value estimation and selection index construction, enables the achievement of genetic gain in populations, optimal resource allocation, and dynamic adjustments of breeding objectives [26].

In this study, both blupADC and DMU employed identical fixed- and random-effect structures when performing mixed-model analyses. Specifically, both incorporated the same fixed-effect variables, such as sex, age, and parity, selected based on prior research and the biological significance inherent in the data, with the aim of controlling for the influence of these known environmental factors on the traits. Simultaneously, both considered the same random-effect structure, including individual direct genetic effects and permanent environmental effects, to assess genetic variation and non-genetic differences among individuals. However, subtle differences exist in the variance component estimates between blupADC and DMU. For instance, the genetic variance component estimated by blupADC was slightly higher than that estimated by DMU, potentially stemming from blupADC’s use of different convergence criteria or optimization algorithms during the iterative process. However, these differences are primarily attributable to variations in algorithms and numerical implementations. Through detailed comparative analysis and sensitivity analysis, we confirmed that these discrepancies have a limited impact on the research conclusions, showing no substantive effect on the core findings.

In this study, the weights of Huaxi cows at different ages were measured and analyzed in detail [27]. The results showed that the birth weight of Huaxi cows ranged between 35 and 45 kg, the 6-month weight was between 160 and 200 kg, the 12-month weight was mostly between 260 and 320 kg, and the adult weight ranged from 500 to 600 kg [28]. These data provide an important reference for understanding the growth and development patterns of Huaxi cows. Compared with previous studies, the weight-gain pattern of Huaxi cows is similar to that of beef cattle [29]. Under conditions of adequate nutrition, the weight of Huaxi cows shows an accelerating growth trend at sexual maturity, and the weight gain gradually slows down when they reach developmental maturity. This growth pattern is consistent with the positive correlation between the growth rate and feed utilization efficiency of beef cattle [30]. That is, the higher the energy (feed) utilization efficiency, the faster the growth rate, and the shorter the time required to reach the market weight [31]. Further analysis of genetic correlations showed significant genetic correlations between weights at different ages and between age-specific weights and adult weight in Huaxi cows [32]. Among them, the genetic correlation between 6-month and 12-month weights was the highest, reaching 0.771, indicating that the weights at these two stages are strongly influenced by the same genetic factors. In contrast, the genetic correlation between 12-month weight and adult weight was relatively low, at about −0.05, suggesting that the weights at these two stages may be regulated by different genetic mechanisms [33]. These findings are of great significance for the genetic evaluation and breeding strategies of Huaxi cows. By understanding the genetic relationships between weights at different ages, breeders can more accurately select individuals with ideal growth performance, thereby improving breeding efficiency and promoting the genetic improvement of the Huaxi cow population [34]. In addition, the growth and development patterns of Huaxi cows also indicate the importance of focusing on the nutritional needs of different growth stages in feeding management to fully realize their growth potential [35].

Research demonstrates that parity, birth weight, and season significantly influence body weight across all growth stages in Huaxi cattle, consistent with our findings. The core mechanisms involve the following: (1) Parity directly regulates calf development by modulating dam physiological maturity and nutritional supply capacity—primiparous cows yield lower birth weights due to immature reproductive systems, while multiparous cows (third–fifth parity) optimize energy metabolism and placental function to maximize birth weight [36]. Beyond the fifth parity, declining reproductive hormones and uterine elasticity drive a quadratic relationship (initial increase followed by decrease) in birth weight. (2) Seasonal effects operate through dual pathways: summer heat–humidity suppresses feed intake and induces heat stress, impeding growth, whereas spring–autumn conditions maximize growth potential through favorable climate and forage resources; concurrently, photoperiod changes mediate metabolic and appetitive regulation via circadian rhythms, establishing seasonal growth patterns [37]. As a pivotal initial variable, birth weight’s high environmental sensitivity underpins its long-term effects on subsequent growth, making it a critical indicator for herd health assessment [37]. To address late-gestation nutritional demands, winter management requires concentrate and silage supplementation to offset green forage shortages, establishing foundations for spring calving success, whereas summer heat stress directly reduces birth weight through nutritional deficits [38]. In conclusion, implementing integrated strategies—including seasonally adapted breeding (spring mating/autumn calving), precision nutritional interventions for late-gestation multiparous cows, parity-specific monitoring protocols, and heat stress mitigation systems—optimizes production performance while enabling sustainable husbandry [39].

In summary, parity, season, and birth weight interactively shape the growth trajectory of Huaxi cattle [40]. Calves with high birth weights, when born to multiparous cows during favorable seasons, can maximize their growth potential by leveraging seasonal environmental advantages [41]. This insight holds significant value for optimizing Huaxi cattle farming practices: by scientifically regulating reproductive cycles (e.g., scheduling spring matings for autumn calvings), calves can benefit from abundant autumn forage resources [42]. Simultaneously, enhancing nutritional management of multiparous cows—particularly through precision feeding during late gestation—can significantly increase calf birth weights, thereby establishing a sustainable and efficient production system. Such refined management strategies based on growth patterns can both improve individual production performance and contribute to the sustainable development of China’s beef cattle industry [43]. However, it should be noted that high birth weights may increase dystocia risk. Thus, while aiming to increase birth weights, effective measures should be implemented to prevent and manage dystocia, including intensifying late-pregnancy monitoring, providing scientific calving assistance, ensuring adequate nutrition and exercise to maintain optimal physical condition, thereby reducing dystocia incidence. Implementing appropriate calving management strategies can effectively mitigate this risk [44].

To gain deeper insights into the growth and genetic traits of Huaxi cattle, we conducted a detailed comparison with other Chinese breeds such as Luxi Yellow Cattle and Jinnan Cattle [45]. However, methodological inconsistencies exist across studies regarding experimental designs and measurement protocols, insufficient sample sizes are observed for certain comparative breeds, and standardized management practices for rearing environments (e.g., nutritional levels and housing conditions) remain lacking. These factors collectively may compromise the direct comparability of phenotypic weight data. In this context, it was observed that Huaxi calves weigh 35–45 kg at birth, 160–200 kg at 6 months, 260–320 kg at 12 months, and 500–600 kg at 24 months. Luxi Yellow Cattle grow slowly initially but accelerate later, with birth weights of 30–40 kg, 140–180 kg at 6 months, 220–280 kg at 12 months, and adult weights of 450–550 kg. Jinnan cattle have similar growth trends but different weights: 32–42 kg at birth, 150–190 kg at 6 months, 230–290 kg at 12 months, and adult weights of 480–580 kg. For heritability, Huaxi cattle show medium–high estimates (0.25–0.39) across different growth stages. Luxi Yellow Cattle have slightly lower heritability (0.20–0.33), while Jinnan cattle have a wider variation (0.18–0.36). The high genetic correlation between 6-month and 12-month weights in Huaxi cattle (0.771) suggests strong shared genetic influences. Jinnan cattle have a correlation of 0.68, and Luxi Yellow Cattle 0.62, indicating comparatively weaker genetic effects [46]. Compared with Luxi and Jinnan cattle, Huaxi cattle demonstrate faster early-stage weight gain and higher heritability, implying greater potential for genetic improvement. The high genetic correlation between growth stages also aids early selection and enhances breeding efficiency. However, differences in sample selection, feeding conditions, and data collection across studies mean these comparative results should be used with caution. Future research should involve more direct comparisons between Huaxi and other Chinese cattle breeds. This will facilitate a deeper understanding of Huaxi cattle growth and genetics, providing a sounder basis for their genetic evaluation and breeding programs, and driving their genetic improvement and industrial development.

## 5. Conclusions

Based on the data used in this study, we conclude that early-life body weight—especially at 6 and 12 months—is significantly and positively correlated with adult weight, enabling prediction of mature size, shortening generation intervals, and accelerating genetic progress. Non-genetic factors such as parity, season of birth, and birth weight exert significant effects on weight at all stages; therefore, seasonal feeding regimes and parity-specific nutritional management must be simultaneously optimized. Consequently, a composite selection index centered on 12-month weight and integrating early weights with morphometric measurements can sustain continuous improvement in growth performance while maintaining genetic diversity. Future work should incorporate genomic information to refine breeding-value accuracy, validate the strategy across diverse ecological zones, and extend the model to multi-trait improvement—including meat quality and fertility—to support efficient, precision breeding across the entire Huaxi cattle industry chain.

## Figures and Tables

**Table 1:** Records from the data screening phase.

Processing Stage	Pre-Processing Records	Removed/Imputed Records	Reason for Removal/Imputation	Post-Processing Records	Cumulative Removal Rate (%)
Initial Raw Data	3637	-	-	3637	0
Remove Records Missing Key IDs	3637	95	Missing ear tags	3542	0.026
Remove Records Missing Core Vars	3542	83	Missing parity/birth year	3459	0.049
Remove High-Missing Records (>30%)	3459	94	Excessive missing core fields	3365	0.075
Remove Duplicate Records	3365	112	Full duplicates (ear tag-based)	3253	0.106
Mean Imputation (Numerical Vars)	3253	159	Imputed weight/chest girth/body length	3094	0.149
Identify and Remove Invalid Outliers	3094	102	Confirmed input/measurement errors	2992	0.177
Final Cleaned Dataset	-	-	-	2992	0.177

**Table 2:** The definition of monthly-age weight and adult weight traits in Huaxi cows.

Item	Abbreviation	Definition
birth weight, kg	WEI-0	The birth weight of Huaxi cows
weight at 6 months of age, kg	WEI-6	The weight of Huaxi cows at six months of age
weight at 12 months of age, kg	WEI-12	The weight of Huaxi cows at twelve months of age
adult weight, kg	WEI	The adult weight of Huaxi cattle

**Table 3:** Descriptive statistics of adult weight, age-specific weight traits, and adult phenotypes in Huaxi cows.

Trait *	No. Records	Mean	SD	CV	Min	Max
WEI-0 (kg)	2992	44.60	6.12	13.90%	23.80	63.87
WEI-6 (kg)	2992	226.56	27.12	12.17%	140.32	360.11
WEI-12 (kg)	2992	357.15	28.10	7.8%	213.31	483.30
WEI (kg)	2992	575.20	36.11	6.30%	421.32	788.10
BH (cm)	2992	124.22	6.10	4.34%	156.19	250.17
CC (cm)	2992	176.17	10.00	5.10%	125.67	296.91
BL (cm)	2992	148.41	18.00	10.10%	128.22	240.76
CH (cm)	2992	21.45	9.11	6.10%	17.19	23.75
SC (cm)	2992	237.11	1.15	4.14%	155.21	260.22
AG (cm)	2992	44.11	10.15	4.41%	5.03	63.08

* WEI-0, Birth weight; WEI-6, Weight at 6 months of age; WEI-12, Weight at 12 months of age; WEI, Adult weight; BH, Body height; CC, Chest circumference; BL, Body length; CH, Cannon circumference; SC, Shoulder circumference; AG, Abdominal girth; SD, Standard deviation; CV, Coefficient of variation.

**Table 4:** Effects of non-genetic factors on age-specific and adult weight traits in Huaxi cows.

Effect	Level	No. Records	WEI-0	WEI-6	WEI-12	WEI *
Parity	1	230	34.32 ± 0.01 ^E^	180.33 ± 0.01 ^E^	240.11 ± 0.01 ^E^	521.62 ± 0.02 ^E^
	2	1188	40.48 ± 0.01 ^D^	223.81 ± 0.01 ^D^	280.18 ± 0.01 ^D^	558.31 ± 0.03 ^D^
	3	1256	42.36 ± 0.02 ^C^	280.90 ± 0.01 ^C^	350.35 ± 0.03 ^C^	652.11 ± 0.03 ^C^
	4	212	45.81 ± 0.03 ^B^	310.76 ±0.03 ^B^	380.98 ± 0.05 ^B^	700.43 ± 0.06 ^B^
	5 and above	106	50.38 ± 0.02 ^A^	320.32 ± 0.02 ^A^	420.77 ± 0.02 ^A^	750.86 ± 0.07 ^A^
Test season	Spring	212	45.32 ± 0.01 ^A^	316.61 ± 0.05 ^A^	362.32 ± 0.03 ^B^	659.23 ± 0.04 ^A^
	Summer	1132	35.48 ± 0.01 ^C^	292.70 ± 0.03 ^B^	300.77 ± 0.07 ^D^	600.10 ± 0.03 ^D^
	Fall	1241	41.36 ± 0.05 ^B^	303.53 ± 0.02 ^C^	320.18 ± 0.02 ^C^	621.56 ± 0.03 ^B^
	Winter	830	40.81 ± 0.03 ^B^	170.11 ± 0.03 ^D^	309.01 ± 0.05 ^E^	610.12 ± 0.03 ^C^
Birth weight	<30 ≥ 30 ≤ 35 ≥ 35 ≤ 40 ≥ 40 ≤ 45 ≥ 45 ≤ 50 ≥ 50 ≤ 55 ≥ 55 ≤ 60	20	1	201.55 ± 0.05 ^G^	223.39 ± 0.02 ^G^	450.48 ± 0.05 ^G^
		50	1	230.32 ± 0.01 ^F^	250.65 ± 0.02 ^F^	501.32 ± 0.03 ^F^
		763	1	261.88 ± 0.03 ^E^	287.76 ± 0.05 ^E^	555,09 ± 0.04 ^E^
		892	1	301.11 ± 0.04 ^D^	307.65 ± 0.03 ^D^	603.41 ± 0.03 ^D^
		125	1	312.98 ± 0.03 ^C^	330.31 ± 0.01 ^C^	652.97± 0.04 ^C^
		89	1	319.55 ± 0.04 ^B^	367.99 ± 0.03 ^B^	702.11 ± 0.05 ^B^
		55	1	325.00 ± 0.05 ^A^	415.05 ± 0.02 ^A^	750.43 ± 0.03 ^A^

* WEI, adult weight; WEI-0, birth weight; WEI-6, weight at 6 months of age; WEI-12, weight at 12 months of age; In the same column of the same factor, values without the same capital letters mean significant difference (*p* < 0.05), while the same capital letter means no significant difference (*p* > 0.05).

**Table 5:** Estimates of additive genetic variance (V^2^), additive genetic variance standard error (V^2^_SE), heritability (h^2^), and heritability standard error (h^2^_SE) for age-specific and adult weight traits in Huaxi cows.

Trait *	No. Records	h^2^	h^2^_SE	V^2^	V^2^_SE
WEI-0	2992	0.25101	0.13262	2.80513	1.88823
WEI-6	2992	0.19545	0.26355	1.65059	1.28745
WEI-12	2992	0.399654	0.15832	2.38922	2.58433
WEI	2992	0.23997	0.13177	1.39436	3.89828

* WEI, adult weight; WEI-0, birth weight; WEI-6, weight at 6 months of age; WEI-12, weight at 12 months of age.

**Table 6:** Estimates of genetic correlation coefficients between different monthly weights and adult weight in Huaxi cows.

	WEI-0	WEI-6	WEI-12	WEI
WEI-0	1	0.298 ^&&^	0.779 ^&^	0.355 ^&&^
WEI-6	0.769	1	0.991 ^&^	0.228 ^&&^
WEI-12	0.770	0.771	1	0.253 ^&^
WEI	0.696	0.762	0.671	1

WEI, adult weight; WEI-0, birth weight; WEI-6, weight at 6 months of age; WEI-12, weight at 12 months of age. Note: The lower triangle is genetic correlation, and the upper triangle is phenotypic correlation; ^&^ and ^&&^ mean *p* < 0.05 and *p* < 0.01, respectively.

## Data Availability

Restrictions apply to the availability of these data. Data were obtained from a commercial dairy farm and are available from the author, Mengli Han, with the permission of Huaxi Dairy Farm.
